# Improving the effects of salt stress by β-carotene and gallic acid using increasing antioxidant activity and regulating ion uptake in *Lepidium sativum* L.

**DOI:** 10.1186/s40529-022-00352-x

**Published:** 2022-07-16

**Authors:** Marziyeh Babaei, Leila Shabani, Shahla Hashemi-Shahraki

**Affiliations:** 1 Department of Biology, Faculty of Science, University of Shahrekord, Shahrekord, Iran; 2 Department of Biology, Faculty of Science, University of Sistan and Baluchestan, Zahedan, Iran

**Keywords:** Antioxidant, β-Carotene, Gallic acid, *Lepidium sativum*, Sodium chloride

## Abstract

**Background:**

Plant growth and development are severely affected by soil salinity. This study was carried out to evaluate the interaction of foliar application of antioxidants (β-carotene and gallic acid) and salt stress on *Lepidium sativum* seedlings.

**Results:**

Our findings revealed that total dry and fresh weight were adversely affected by 25 mM NaCl salinity stress. Moreover, K^+^ content decreased while Na^+^ content increased significantly. The foliar application of β-carotene and gallic acid significantly mitigated the effects of salt stress by regulating ion uptake, reducing H_2_O_2_ and malondialdehyde (MDA) content, as well as increasing enzymatic antioxidant activity, phenolic, glutathione, and chlorophyll content.

**Conclusions:**

β-carotene- and gallic acid-treated plants had higher salt tolerance.

## Introduction

A plant’s secondary metabolites are not essential for completing the plant growth and development cycle. Still, they play an important role in regulating plant interactions and their adaptation to environmental factors, as well as in defense processes against biotic and abiotic stresses (Yang et al. 2018). In higher plants, a wide range of secondary metabolites is synthesized from primary metabolites such as carbohydrates, lipids, and amino acids at low concentrations. Environmental factors (e.g., temperature, humidity, light intensity, water supply, minerals, CO_2_) influence the production of secondary metabolites (Akula and Ravishankar 2011; Alvarado et al. 2019).

**Fig. 1:**
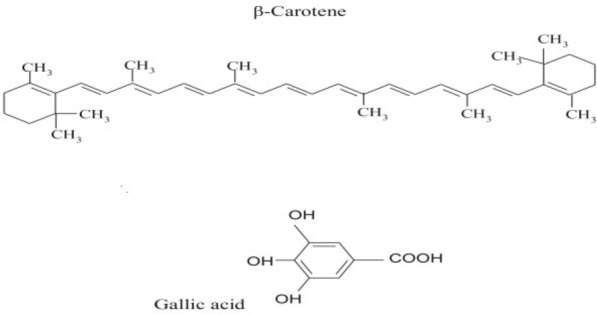
Chemical structures of β-carotene and gallic acid

The main carotenoids in plants are lutein, lycopene, and β-carotene (Fig. 1). β-carotene and other carotenoids are the most efficient natural ^1^O_2_ quenchers (Siems et al. 1999). The number of double bonds in their molecules is closely related to their quenching activity. β-carotene can help quench singlet oxygen and scavenge free radicals. Several studies indicate that plants under environmental stress improve the adverse effects of stress by increasing β-carotene content. For example, Kim et al. (2012) suggested that increased levels of β-carotene in transgenic cultured cells of sweet potato cause stress tolerance to salt. Also, Kim et al. (2012) reported that high salt concentrations increased antioxidant capacity in tomatoes and increased the carotene content by 35%. Long-term salt treatment in lettuce increases β-carotene and lutein levels by up to 80 and 37%, respectively (Kim et al. 2008). Like β-carotene, gallic acid is a crucial plant antioxidant. Gallic acid is a component of phenolic compounds (Fig. 1). Gallic acid is an effective compound in scavenging free radicals and inhibiting the lipid peroxidation process. It also improves the effects of various abiotic stresses such as chilling, heavy metals, and osmotic stress. Yildiztugay et al. (2017) reported that gallic acid treatment led to higher resistance in soybean plants to cold stress via its ability to scavenge free radicals, improve water status, and photosynthetic capacity. Treatment of gallic acid in tomato callus reduces the negative effect of excessive boron-induced damage by reducing boron uptake or preventing growth inhibition (Farghaly et al. 2021). However, very little research is available on the exogenous use of β-carotene and gallic acid in reducing the adverse effects of salinity stress on plants. The main purpose of this study was to investigate the impact of exogenous gallic acid and β-carotene on *Lepidium sativum* exposed to salt.

## Materials and methods

Preliminary experiments were carried out to determine the threshold concentration of salinity (which caused the lowest detrimental effect) and the most effective concentration of phenolic compounds on *L. sativum* growth parameters. In the preliminary experiments, the effects of different concentrations of NaCl (0, 25, 50, 100, 150, 200, 250, 300 and 350 mM), β-carotene (0, 0.5, 2.5 and 5 mM) and gallic acid (0, 0.5, 1, 2.5, 5, 10, 12.5, 15 and 20 mM) were evaluated on seed germination and seedling growth for 5 days. According to the findings of the preliminary experiments, 25 mM NaCl salinity, 0.5 mM β-carotene, and 5 mM gallic acid were chosen for the main experiment (Data not shown, Figure Suppl number 1–13).

The main experiment was carried out in a completely randomized design with three replications. Ten surface-sterilized seeds were placed in the 90 mm petri dish containing 30 ml of distilled water and incubated in a growth chamber (25 °C, 50% RH, 16/8 h day/night photoperiod, 120 µmol m^−2^ s^−1^ light intensity) for 5 days. Five-day-old seedlings were transferred to culture trays containing perlite and fertigated with 1/2 Hoagland solution (Peralta-Videa et al. 2002). The seedlings were transferred to a greenhouse (16/8 h day/night photoperiod, 27 °C). β-carotene (0.5 mM) and gallic acid (5 mM) solutions were sprayed on the seedlings for three consecutive days (three times a day). After antioxidant treatment, 8-day-old seedlings received 1/2 Hoagland solution containing 25 mM NaCl.

Treatments included: (1) Control (2) plants sprayed with β-carotene (3) plants sprayed with gallic acid (4) plants received 25 mM NaCl (5) plants sprayed with β-carotene and received 25 mM NaCl (6) plants sprayed with gallic acid and received 25 mM NaCl.

Fifteen-day-old seedlings were harvested, and their fresh (FW) and dry weights (DW) were measured and then stored at − 80 °C for further analysis.

### Determination of total chlorophyll content

The total chlorophyll content was determined spectrophotometrically using 0.1 g FW of leaf tissue ground with mortar and pestle in 10 ml of acetone 80% (v/v). After centrifugation and reading the absorbance values at 663 and 645 nm, the values in the following equations were used (Arnon 1949). The contents were expressed as mg total chlorophyll g^−1^ FW.

1 $$Total \, chlorophyll \left( {mg/gFW} \right) = \left[ {\left( {8.02 \times A663} \right) + \left( {20.2 \times A645} \right)} \right] \times \frac{{volume \, of \, acetone\; \left( {ml} \right)}}{{weight \, of \, sample \;\left( {mg} \right) \times 1000}}$$

### Determination of total phenolic content and DPPH radical scavenging activity

Using the Singleton and Rossi (1965) methods, the total soluble phenolic compounds were estimated. The frozen leaf tissue (0.1 g) was ground with 3 ml of 80% methanol in a cooled mortar. The obtained extract was centrifuged at 15,000 rpm for 15 min. The supernatant was used to measure the amount of phenolic compounds. The absorbance of the reaction mixture consisting of 30 μl of extract, 120 μl of sodium carbonate (Na_2_CO_3_), and 150 μl of Folin–Ciocalteu reagent was read at 765 nm after exposure to darkness for 30 min.

Utilizing the Kulisic et al. (2004) method, radical scavenging activity was estimated by a spectrophotometric method based on the reduction of a methanol solution of 2,2‐diphenyl‐1‐picryl‐hydrazyl‐hydrate (DPPH). An aliquot of 1 ml of the plant extract was added to 1 ml DPPH solution in a concentration of 100 µM in methanol. The control sample was prepared without any extract. The absorbance was measured at 517 nm, after 30 min incubation in darkness at ambient temperature. Radical scavenging activity was calculated using the following equation.

2 $$Inhibition \left( \% \right) = \frac{A\,control - A \, sample}{{A \, control}} \times 100$$

### Measurement of oxidative stress markers

#### Determination of H_2_O_2_ and malondialdehyde (MDA) content

H_2_O_2_ was measured spectrophotometrically (λ = 390 nm) by a reaction with 1 M KI according to Alexieva et al. (2001). 0.1 g of frozen leaf tissue was ground in a cooled mortar with 1.5 ml of TCA (trichloroacetic acid) 0.1%. The obtained extract was centrifuged at 15,000*g* for 4 min at 4 °C. Five hundred μl of supernatant was added to 500 μl of 10 mM phosphate buffer and 1 ml of 1 M KI solution. The absorption rate was read by a spectrophotometer at 390 nm. The malondialdehyde (MDA) content was detected by commercially available kits (KIA ZIST, Hamadan, Iran). The MDA content was expressed as nmol MDA/gFW.

#### Determination of glutathione (GSH) level, catalase (CAT), superoxide dismutase (SOD) guaiacol peroxidase (GPX), glutathione reductase (GR) and ascorbate peroxidase (APX) enzymes activities

Glutathione (GSH) level and glutathione reductase (GR) were detected by commercially available kits (KIA ZIST, Hamadan, Iran). The GSH levels and GR activity were measured according to the manufacturer’s instructions. The absorption of both was measured using a spectrophotometer at 405 nm.

The CAT, SOD and GPX, APX enzyme activities were determined by the following method. 0.1 g of each sample was homogenized in the extraction phosphate buffered saline (PBS) pH 7.8. The catalase activity was measured utilizing the method described by Aebi (1984). The activity was estimated via monitoring the decrease in absorbance due to H_2_O_2_ decomposition [extinction coefficient (ε = 39 µmol^−1^ cm^−1^)] at 240 nm. The reaction mixture contained 50 µl plant extract, 50 mM phosphate buffer (pH 7.0), and 10 mM H_2_O_2_. The SOD activity was measured spectrophotometrically, as described by Beyer and Fridovich (1987), and assayed by monitoring the inhibition of photochemical reduction of nitro blue tetrazolium (NBT). For the amount of enzyme which causes 50% inhibition of the NBT photo-reduction rate in 1 min at 560 nm, a unit of SOD activity was defined. The specific activity of SOD was expressed as unit/mg FW. For GPX activity estimation (Lin and Kao 1999), the reaction was initiated by adding the plant extract (50 µl) to a reaction mix comprising 50 mM phosphate buffer, 19 mM H_2_O_2,_ and 9 mM guaiacol. The absorbance was recorded at 470 nm for the amount of enzyme which forms 1 μM tetraguaiacol per minute at 470 nm. According to the Nakano and Asada (1987) method for the measurement of APX activity, 50 µl plant extract, 50 mM phosphate buffer containing 50 mM phosphate buffer comprising 0.5 mM ascorbic acid and 0.25 M H_2_O_2_ were utilized. Using a decrease in adsorption at 290 nm, hydrogen peroxide-dependent oxidation of ascorbate was followed.

#### Determination of Na^+^ and K^+^ concentration

The ash sample of shoots and roots were acid digested and filtered to determine Na^+^ and K^+^ concentration by a flame photometer (Corning, UK). Na^+^ and K^+^ content were recorded in mg/g DW.

#### Statistical analysis

Data was analyzed using SPSS ver. 17.0 software and means were compared using Duncan test at 5% probability level.

## Results

**Fig. 2:**
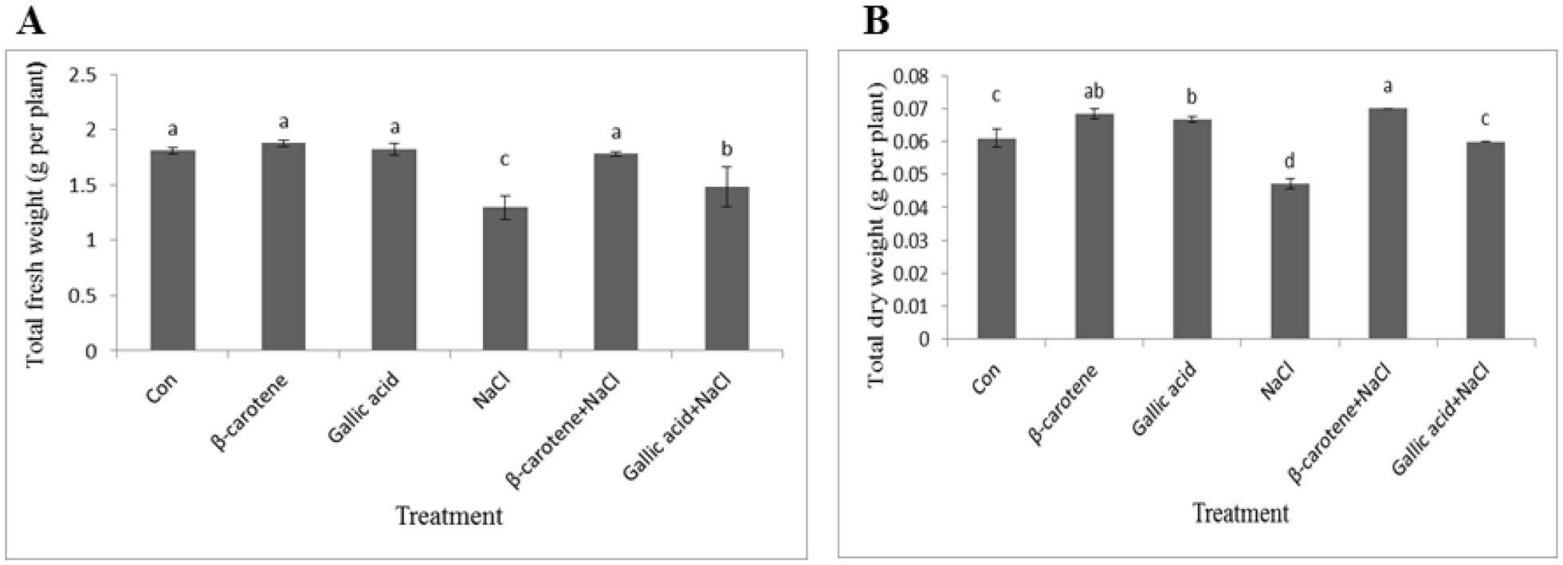
Effect of β-carotene and gallic acid, under saline and non-saline conditions on the total fresh/dry weight in *Lepidium sativum* seedlings. Columns with the same letters are not statistically different at 5% probability using Duncan’s test

β-carotene or gallic acid (alone) did not change fresh weight. Salinity stress caused 28% reduction in fresh weight. In plants sprayed with β-carotene salinity stress did not decreased this parameter in comparison to control samples. Foliar application of gallic acid mitigated the effect of salinity as treated plants had significantly higher fresh weight compared to non-treated, salt-stressed plants (Fig. 2A).

Foliar application of β-carotene and gallic acid caused an increase in dry weight of plants under non-saline condition. Salt stress resulted in 22% reduction in total dry weight compared to the control. Salt−stressed plants sprayed with β-carotene had higher dry weight compared to the control. In salt-stressed plants sprayed with gallic acid this parameter did not change significantly (Fig. 2B).

**Fig. 3:**
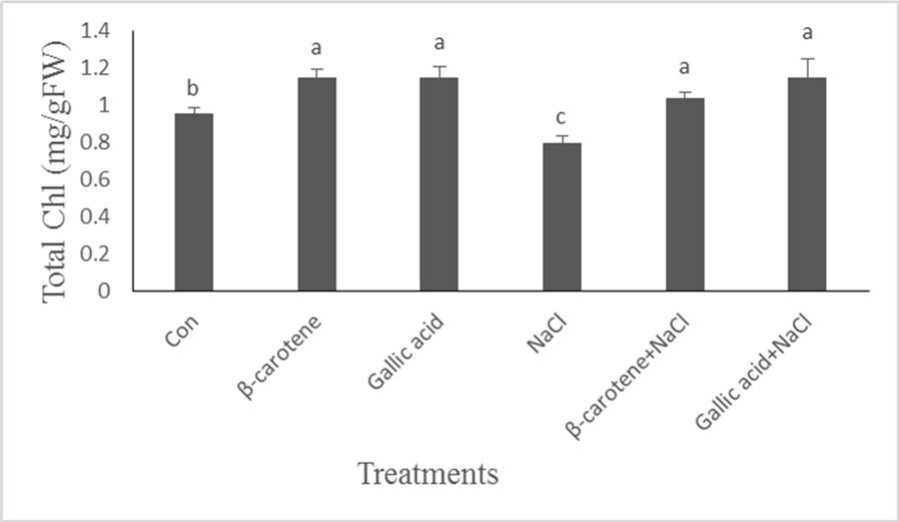
Effect of β-carotene and gallic acid, under saline and non-saline conditions on the total chlorophyll concentration in *Lepidium sativum* seedlings. Columns with the same letters are not statistically different at 5% probability using Duncan’s test

Salinity had a negative effect on total chlorophyll content, whereas pretreatment with β-carotene and gallic acid increased total chlorophyll concentration under non-saline conditions. Under the saline condition, the foliar application of β-carotene or gallic acid caused an increase in total chlorophyll concentration (35 and 44%, respectively) compared to salt-stressed plants (Fig. 3).

**Fig. 4:**
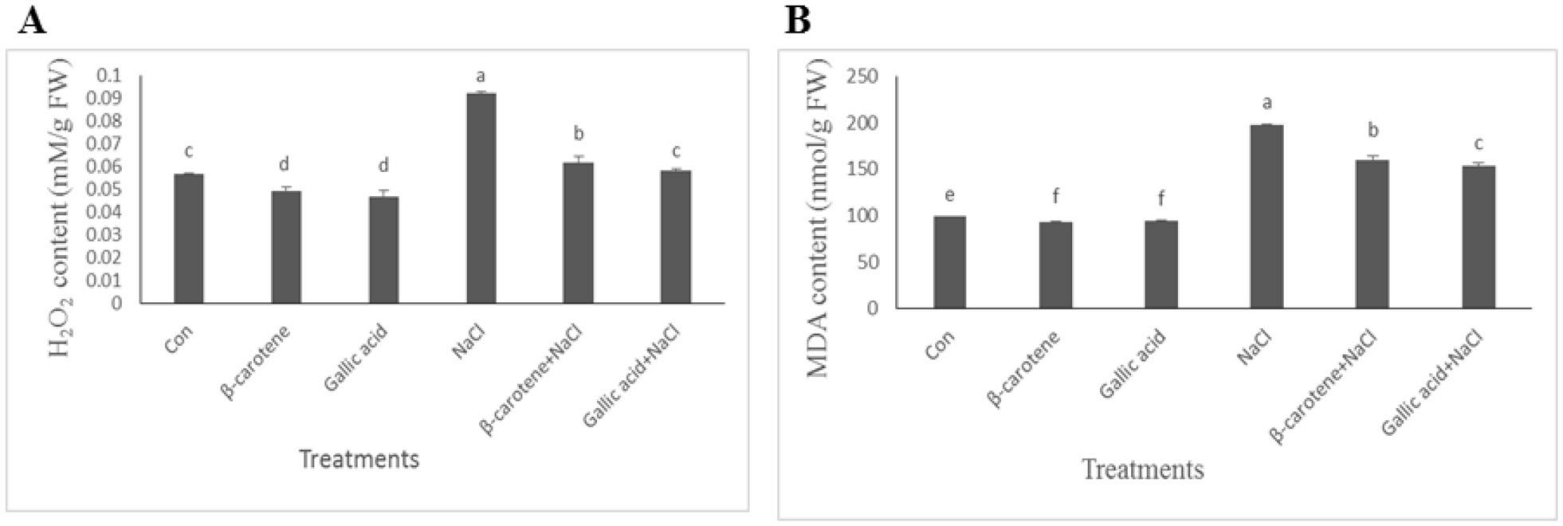
Effect of β-carotene and gallic acid, under saline and non-saline conditions on H_2_O_2_ concentration (**A**) and MDA content (**B**) in *Lepidium sativum* seedlings. Columns with the same letters are not statistically different at 5% probability using Duncan’s test

In plants pretreated with β-carotene and gallic acid, H_2_O_2_ content decreased by 13% and 17%, respectively, compared with the control under non-saline conditions. Salinity increased H_2_O_2_ content by 63% compared with the control samples. Treatments with β-carotene + sodium chloride and gallic acid + sodium chloride decreased H_2_O_2_ content by 33 and 37%, respectively, compared with the salinity-stressed plants (Fig. 4A).

Compared with the control samples, MDA concentration decreased by 6 and 5% in plants sprayed with β-carotene and gallic acid. Salinity caused a 99% increase in MDA concentration. Treatments with β-carotene and gallic acid decreased MDA content by 19 and 22% under saline conditions compared with the salt-stressed plants (Fig. 4B).

**Fig. 5:**
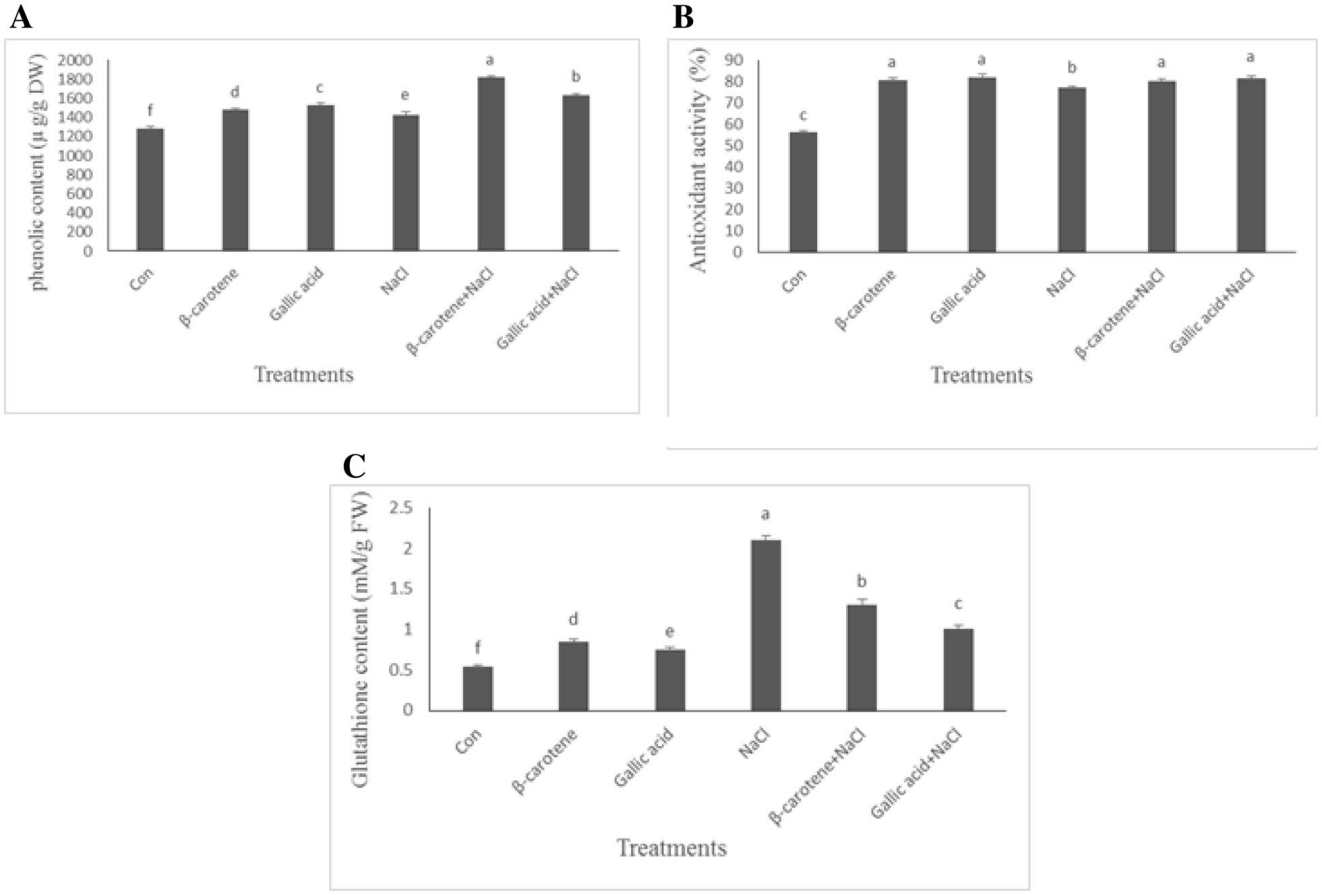
Effect of β-carotene and gallic acid, under saline and non-saline conditions on the phenolic content (**A**), glutathione content (**B**), and DPPH radical scavenging activity (**C**) in *Lepidium sativum* seedlings. Columns with the same letters are not statistically different at 5% probability using Duncan’s test

Phenolic compounds content increased by 15, 19, and 11%, respectively in plants received β-carotene, gallic acid, and sodium chloride treatments. β-carotene and gallic acid treatments increased phenolic compounds by 28 and 15%, respectively, under saline conditions compared to the salt-stressed plants (Fig. 5A).

The antioxidant activity of the plant received β-carotene, gallic acid, and sodium chloride increased by 44, 46, and 38%, respectively, compared with the control samples. β-carotene + sodium chloride and gallic acid + sodium chloride increased the antioxidant activity of plants by 4 and 6%, respectively, compared with the salt-stressed plants (Fig. 5B).

Glutathione concentration in plants treated with β-carotene and gallic acid increased by 56 and 37%, respectively, under non-saline conditions. Salt-stressed plants had 3.84-fold higher glutathione levels compared with control samples. Under saline conditions, β-carotene and gallic acid treatments decreased this parameter by 38 and 52%, respectively (Fig. 5C).

**Fig. 6:**
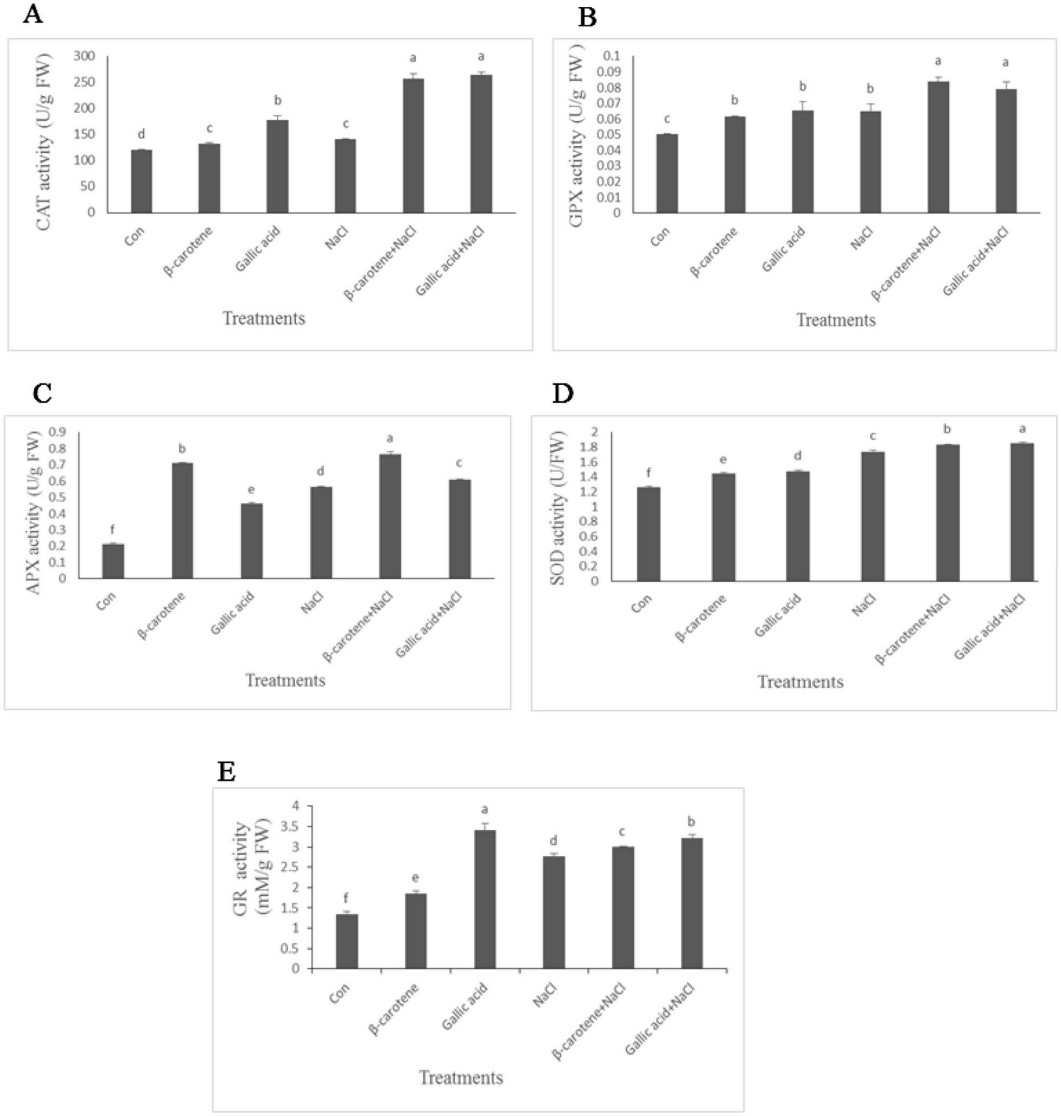
Effect of β-carotene and gallic acid, under saline and non-saline conditions on the activity of CAT (**A**), GPX (**B**), APX (**C**), SOD (**D**), GR (**E**) in *Lepidium sativum* seedlings. Columns with the same letters are not statistically different at 5% probability using Duncan’s test

Activity of enzymatic antioxidants, including CAT, GPX, APX, SOD, and GR increased in the salt-stressed plants. β-carotene and gallic acid treatments alone significantly increased the activity of CAT, GPX, APX, SOD, and GR enzymes compared with control samples. In plants received gallic acid the activity of CAT, SOD, GR was higher in comparison with plants sprayed with β-carotene under non-saline conditions. β-carotene and gallic acid under saline conditions significantly increased the activity of CAT, GPX, APX, SOD, and GR enzymes; however, the increase in the activities of SOD, GR were higher for gallic acid treatment compared with the β-carotene treatment under saline conditions. (Fig. 6A–E).

**Fig. 7:**
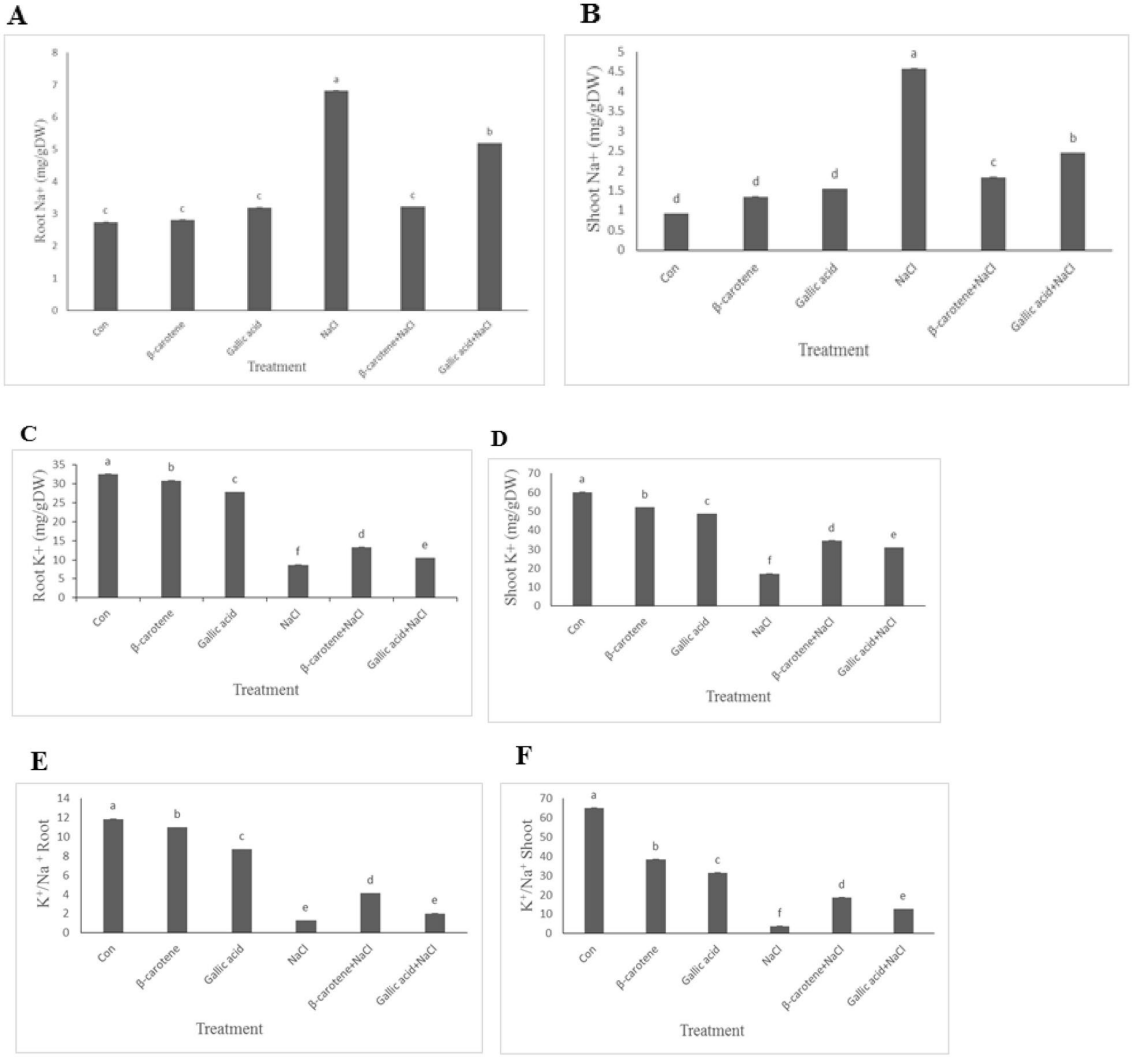
Effect of β-carotene and gallic acid, under saline and non-saline conditions on Na^+^ concentration in roots (**A**), shoots (**B**), K^+^ concentration in roots (**C**), K^+^ in shoots (**D**), K^+^/Na^+^ ratio in roots (**E**), K^+^/Na^+^ ratio in shoot (**F**) in *Lepidium sativum* seedlings. Columns with the same letters are not statistically different at 5% probability using Duncan’s test

β-carotene and gallic acid did not change shoot and root Na^+^ concentration. Salt stressed plants had higher shoot and root Na^+^ concentration. Plants sprayed with β-carotene or gallic acid had significantly lower shoot and root Na^+^ concentration under saline conditions. β-carotene and gallic acid foliar application as well as salinity caused a reduction in shoot and root K^+^ concentration. This parameter was significantly higher in plants sprayed with β-carotene or gallic acid under saline conditions, in comparison with the salt-stressed plants. Similar pattern was observed in K^+^/Na^+^ ratio in shoots and roots (Fig. 7A–F).

## Discussion

According to our results, β-carotene and gallic acid played an essential role in improving the growth of *Lepidium sativum* seedlings under salt stress in terms of increasing biomass production. Compared with the control, pretreatment with β-carotene and gallic acid improved total fresh and dry weight under saline and non-saline conditions. Our results showed that β-carotene was more effective than gallic acid in mitigating salt stress, while both antioxidants had similar effects under non-stress conditions. Similarly, Dawood et al. (2019) reported that β-carotene treatment significantly increased sunflower plants’ fresh weight and shoot length under drought stress. The improvement of growth parameters by the application of β-carotene and gallic acid under salt stress is associated with a decrease in Na^+^ uptake; decreased Na^+^ uptake results in a higher K^+^/Na^+^ ratio. A well-known strategy to counteract Na^+^ is to increase K^+^ intake. A balanced K^+^/Na^+^ ratio is vital for photosynthesis, protein synthesis and activation of numerous enzymes, stomatal function, and adjustment of cell osmoregulation (Zheng et al. 2008). According to our findings, Saleh and Madany (2015) reported that coumarin treatment significantly improved the K^+^/Na^+^ ratio in wheat seedlings under stress and non-stress conditions. Wakeel et al. (2011) found that Na^+^ toxicity affects the reduction of the K^+^/Na^+^ ratio, and consequently, lower K^+^ concentration induced by higher Na^+^ in plant cells affects plasma membrane (PM) H^+^-ATPase activity.

Salinity stress caused a decrease in total chlorophyll concentration while β*-*carotene and gallic acid ameliorated salinity stress. Similar to our findings, exogenously applied caffeic acid alleviated the adverse effects of salinity stress in soybean and improved chlorophyll content (Klein et al. 2015). Wu et al. (2015) suggested that β*-*carotene can protect the photosynthesis apparatus of transgenic tobacco plants under salt stress. An accurate indicator of abiotic tolerance in plants is the chlorophyll content. Increased activity of chlorophyll-degrading enzymes and inhibition of chlorophyll biosynthesis due to increased ethylene production under salt stress conditions result in lower chlorophyll content. An increase in chlorophyll content in plants treated with β-carotene and gallic acid could be due to ROS scavenging properties of β-carotene and gallic acid and an increase in the activity of enzymatic antioxidants. Wu et al. (2015) suggested that the antioxidant effect of β-carotene is related to the biosynthesis of zeaxanthin, a biochemical derivative of β-carotene formed by the activity of the enzyme β-carotene hydroxylase (BCH) by attaching the hydroxyl group to each of the β-carotene rings (Zhu et al. 2008). An important component of the xanthophyll cycle is zeaxanthin, which plays a key role in scavenging reactive oxygen species (ROS) and non-photochemical quenching. In addition to its antioxidant function, β-carotene is able to bind to photosystems I (PSI) and II (PSII) in the reaction center subunits and assist lutein and other xanthophylls in absorbing excess light energy used to transfer photosynthetic electrons (Cazzaniga et al. 2016).

Treatment with β-carotene and gallic acid under non-stress and salt stress conditions increased phenolic compound content in our study. Similar to our results, phenolic compounds increased in cucumber plants under salt stress (Hýsková et al. 2017). In rice plants treated with rutin and gallic acid, this parameter was about 1.4 and 1.31 times higher than in control samples (Singh et al. 2017). Phenolic compounds act as radical scavengers by donating electrons or hydrogen atoms. Under natural conditions, there is a balance between the production and quenching of ROS. Under stress conditions such as salinity, the production of ROS is higher than the quenching, resulting in oxidative stress. The accumulation of ROS causes lipid peroxidation of the membrane, decreasing membrane fluidity and selectivity. Lipid peroxidation causes the formation of malondialdehyde (MDA), which is considered a sign of oxidative damage (Juknys et al. 2012). To mitigate the adverse effects of ROS, plants have an enzymatic antioxidant system including superoxide dismutase (SOD), catalyzing the conversion of O_2_ to H_2_O_2_, which in turn is degraded by the combined activities of catalase (CAT), guaiacol peroxidases (GPX), and ascorbate–glutathione cycle enzymes (Kubiś 2008). Salinity stress in *L. sativum* increased H_2_O_2_ concentration, whereas antioxidant treatments, especially gallic acid, reduced H_2_O_2_ content. H_2_O_2_ could be detoxified by the activity of CAT, GPX, APX, and GR, as well as by the ascorbate–glutathione cycle. Molecular hydrogen peroxide is relatively stable, and its reaction with most biological molecules is limited (Ledo et al. 2022). H_2_O_2_ is electrically neutral ROS, but it is detrimental because it can cross cell membranes and reach cell parts far from the site of its formation. Ozfidan-Konakci et al. (2019) reported that cold stress significantly increased H_2_O_2_ content in soybean roots. They found that gallic acid can contribute to the detoxification of H_2_O_2_ by increasing the activity of the enzymes CAT and APX. Singh et al. (2017) also showed that H_2_O_2_ content decreased in plants treated with gallic acid, with the control plants having the highest amount of H_2_O_2_. APX and GR are the glutathione-ascorbate cycle enzymes. APX is considered the major H_2_O_2_ scavenging enzyme in plant cells, while GR plays a crucial role in maintaining glutathione depletion during stress. According to our results, β-carotene and gallic acid increased the activity of these enzymes during salt stress.

Glutathione (GSH), γ-l-glutamyl-l-cysteinyl-glycine, is a free radical scavenger and important antioxidant (Zechmann 2014). The vital role of glutathione in regulating and controlling the intracellular oxidative balance is attributed to the thiol group. Oxidation/reduction of the thiol group leads to the formation of glutathione disulfide (GSSG) and reduced glutathione (GSH), respectively. GSSG is reduced to GSH by GR (Zechmann 2014). In this study, salt stress significantly increased glutathione levels. The use of β-carotene, gallic acid, β-carotene + NaCl, and gallic acid + NaCl also increased glutathione content. Increased glutathione content under salt stress or after application of gallic acid and β-carotene could be related to increased GR activity. An increased GSH content and higher activity of GR under salinity may be a viable mechanism to maintain a balanced antioxidant state. In contrast to our results, salt stress decreased glutathione content in wheat, whereas β-carotene treatment increased glutathione content, which was in accordance with our findings (Hemida et al. 2014).

High antioxidant activity suggests a possible higher stress tolerance of plants (Sairam et al. 2000). In our experiment, salinity significantly reduced antioxidant activity but was mitigated by β-carotene and gallic acid. This is in agreement with Egedigwe and Udengwu’s (2021) findings, who reported increased DPPH radical scavenging activity in *Amaranthus hybridus* after treatment with β-carotene. Similarly, Kahveci et al. (2021) found that treatment with β-carotene resulted in an increase in phenolic compounds and DPPH radical scavenging activity in *Ocimum basilicum* under salinity.

## Conclusion

**Fig. 8:**
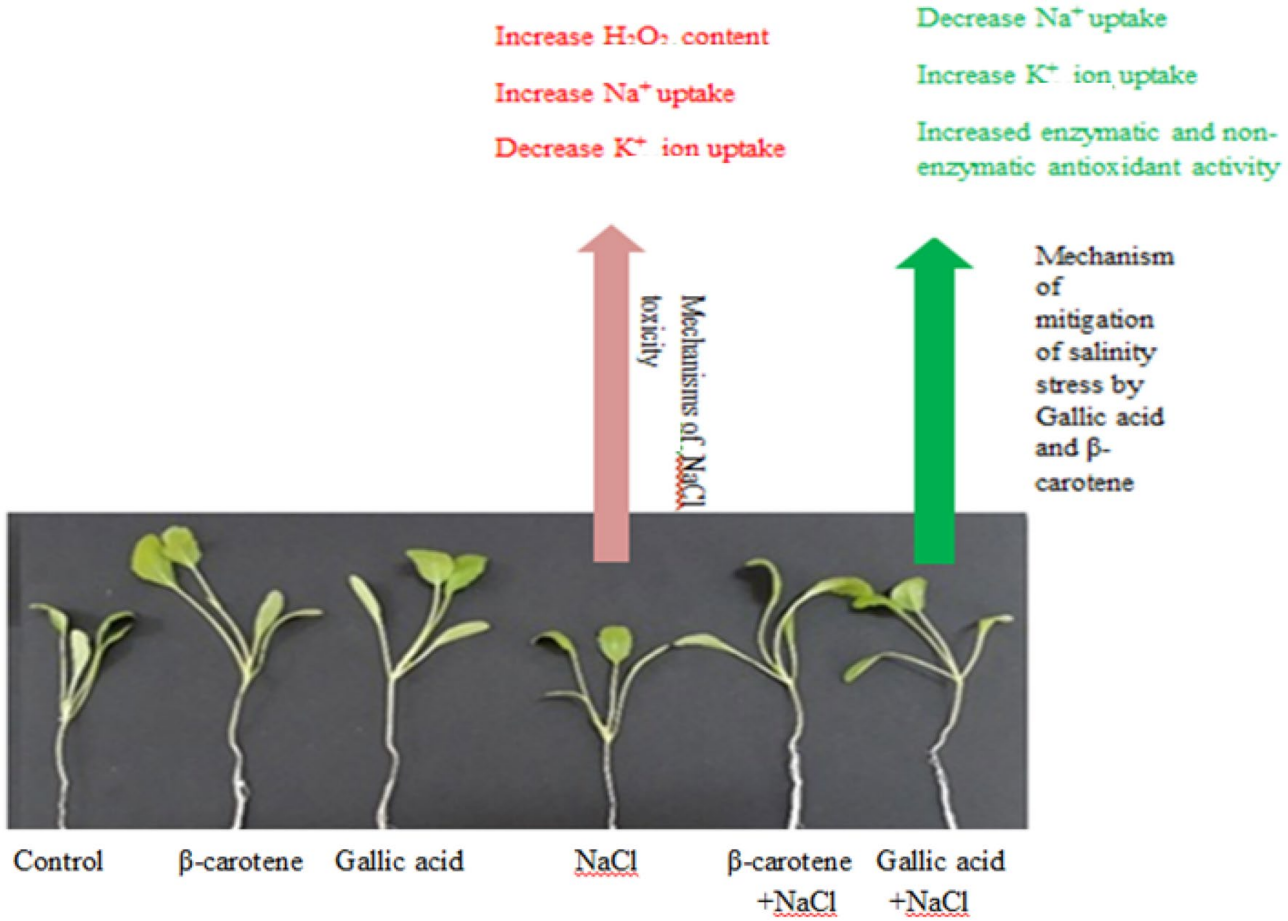
The NaCl detrimental impact and mitigation mechanisms of β-carotene and gallic acid in *Lepidium sativum*

This study showed that salt stress had detrimental effects on *Lepidium sativum*, as confirmed by a reduction in growth and chlorophyll content and an increase in MDA and H_2_O_2_ content. Treatment with β-carotene and gallic acid improved the tolerance of *L. sativum* under salt stress by increasing the growth and content of chlorophyll, glutathione, phenolics, antioxidant activity and by regulating ion uptake (Fig. 8). The process of alleviating salt stress by β-carotene and gallic acid could be similar.

## Abbreviations

MDA: Malondialdehyde
GSH: Glutathione
CAT: Catalase
SOD: Superoxide dismutase
GPX: Guaiacol peroxidase
GR: Glutathione reductase
APX: Ascorbate peroxidase
